# Coastal reclamation alters soil microbial communities following different land use patterns in the Eastern coastal zone of China

**DOI:** 10.1038/s41598-021-86758-2

**Published:** 2021-03-31

**Authors:** Wen Yang, Nasreen Jeelani, Andong Cai, Xiaoli Cheng, Shuqing An

**Affiliations:** 1 College of Life Sciences, Shaanxi Normal University, No. 620 West Chang’an St., Chang’an Dist., Xi’an, 710119 Shaanxi People’s Republic of China; 2 School of Life Sciences and Institute of Wetland Ecology, Nanjing University, Nanjing, 210023 People’s Republic of China; 3 Key Laboratory for Agro-Environment, Ministry of Agriculture, Institute of Environment and Sustainable Development in Agriculture, Chinese Academy of Agricultural Sciences, Beijing, 10081 People’s Republic of China; 4 School of Ecology and Environmental Sciences, Yunnan University, Kunming, 650091 People’s Republic of China

**Keywords:** Ecology, Microbial ecology, Wetlands ecology, Microbial communities

## Abstract

Coastal reclamation seriously disturbs coastal wetland ecosystems, while its influences on soil microbial communities remain unclear. In this study, we examined the impacts of coastal reclamation on soil microbial communities based on phospholipid fatty acids (PLFA) analysis following the conversion of *Phragmites australis* wetlands to different land use types. Coastal reclamation enhanced total soil microbial biomass and various species (i.e., gram-positive bacterial, actinomycete, saturated straight-chain, and branched PLFA) following the conversion of *P. australis* wetland to aquaculture pond, wheat, and oilseed rape fields. In contrast, it greatly decreased total soil microbial biomass and various species following the conversion of *P. australis* wetland to town construction land. Coastal reclamation reduced fungal:bacterial PLFA, monounsaturated:branched PLFA ratios, whereas increasing gram-positive:gram-negative PLFA ratio following the conversion of *P. australis* wetland to other land use types. Our study suggested that coastal reclamation shifted soil microbial communities by altering microbial biomass and community composition. These changes were driven primarily by variations in soil nutrient substrates and physiochemical properties. Changes in soil microbial communities following coastal reclamation impacted the decomposition and accumulation of soil carbon and nitrogen, with potential modification of carbon and nitrogen sinks in the ecosystems, with potential feedbacks in response to climate change.

## Introduction

Coastal wetlands are the transitional zone between terrestrial and marine ecosystems, which provide critical ecosystem services^1^, including biodiversity preservation, flooding and shoreline erosion control, and environmental remediation^2^. In contrast, various anthropogenic activities, particularly coastal reclamation, are altering coastal wetland ecosystems on a global scale^3^. Many Euro-American countries, for instance, the United States and Spain, and Asian countries, such as South Korea and Japan^4^, have reported intensive coastal reclamation operations. The coastal wetlands of China have been dramatically reclaimed for the development of agriculture, aquaculture^3^, urbanization, and industrialization in an attempt to alleviate the conflict between a growing population and limited land resources^5^. The total reclaimed coastal areas in China, from 1950–2008, was approximately 13,380 km^2^^6^, which accounted for approximately half of its overall coastal wetlands. According to the land-use plan of China, 5780 km^2^ of coastal wetlands will be reclaimed from 2010–2020. However, this immensely decreased number of coastal wetland areas following reclamation has been accompanied by multiple negative effects on coastal ecosystems, including a significant reduction in coastal habitats and biodiversity, and disrupted ecosystem structure, processes and function, and has a far-reaching effect on their ecological services^6^.

Coastal wetlands are recognized as one of the vital components of ‘blue carbon (C)’ sinks, as the result of high primary productivity and low decomposition rates of soil organic matter (SOM)^7^. Hence, coastal wetlands play a significant role in the global C cycle. Coastal reclamation can modify the morphologies, hydrodynamics, and sediment transport of coastlines^8^, toward the further alteration of the physicochemical properties of soils^6^. Ultimately, these changes alter soil organic carbon and nitrogen (SOC and SON, respectively) sequestration in coastal wetlands^9^. Although the response of SOC and SON sequestration to coastal reclamation have been widely documented^2,10,11^, there remains no definitive consensus. For example, Ding et al.^10^ revealed that SOC and SON stocks were rapidly sequestered within an initial 50 years following the reclamation of coastal wetlands, and then increased slowly within the reclaimed paddy soil of China's Yangtze River Delta. However, a previous study documented that the sequestration of soil C decreased, while C emissions accelerated, following the conversion of coastal wetlands to farmlands and other land uses^12^. These inconsistent results might have been attributed to variable land use patterns, reclamation intensity, and field management practices^6^.

Soil microbes have vital roles in the regulation and control of soil C and nitrogen (N) cycling^13^. Soil microbial communities are driven by topography, vegetation, soil nutrient substrates^14^, as well as physiochemical properties^15,16^. Soil pH is considered to be one of the vital drivers that shifts the composition of microbial communities^15^. Soil salinity has been observed to exert an inhibitory effect on most microbial populations^17^, where high salinity reduces the osmotic potential of the soil, which further impacts microbial composition and functionality^16^. Soil moisture has been reported to affect microbial abundance/population structures by influencing soil aeration conditions^18^. Yuan et al*.*^19^ demonstrated that high soil aeration stimulated microbial biomass and shifted the composition of microbial communities in paddy fields. High soil aeration can increase the fungi to bacteria ratio in farmlands. Previous investigations revealed that soil nutrient levels changed greatly, soil pH and salinity decreased considerably, and soil aeration was enhanced following the conversion of coastal wetlands to farmlands via diking, ditch drainage, and fresh water irrigation^5,6,20^. Changes in nutrient levels and physiochemical properties strongly drive variations in the microbial biomass and community compositions of soils^14,16^. The alterations in soil microbial biomass and community composition, particularly the fungi to bacteria ratio, in turn, impact the turnover and sequestration of SOC and SON. Therefore, an accurate assessment of the effects of coastal reclamation on soil microbial biomass and community compositions is critical to better understand the influencing mechanisms of coastal reclamation on SOC and SON accumulation and decomposition in coastal wetlands.

**Figure 1:**
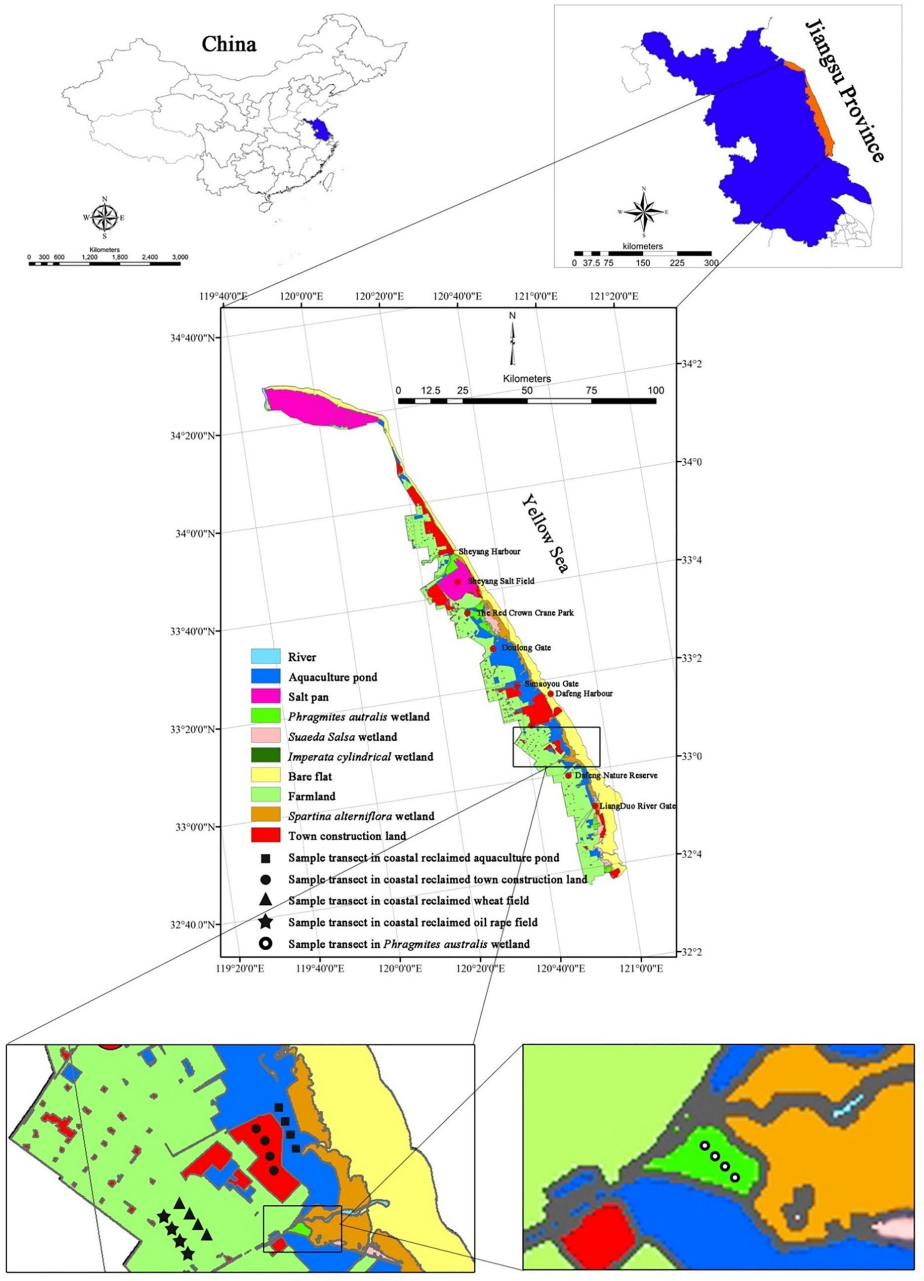
Location of the sampling site in different reclaimed coastal land use types, and a natural *P. australis* wetland in the Eastern coastal zone of China. Figure generated in ArcGIS 9.3. URL link: http://desktop.arcgis.com/zh-cn/desktop/.

Jiangsu province contains the most abundant coastal wetland resources in Eastern China, and is undergoing intense coastal reclamation^4^. Currently, a large portion of the natural coastal wetlands in Jiangsu have been reclaimed by embanking (e.g., construction of dikes, seawalls, and barriers along the coastline), and subsequent conversion to aquaculture ponds, farmlands, and town construction lands (Fig. 1)^4^. Numerous studies have documented the effects of coastal reclamation on ecosystem C and N sinks, especially on SOC and SON sequestration along the coasts of Eastern China^9,11,21^. Our previous study found that coastal reclamation greatly altered soil total, labile and recalcitrant organic C and N following the conversion of coastal wetlands to different reclaimed lands^21^. However, these studies set their focus primarily on the variations of SOC and SON, while their mechanisms of influence on soil microbial ecology received little attention. The responses of soil microbial communities to coastal reclamation, following the conversion of coastal wetland to aquaculture pond, farmlands, and town construction land, and these responses whether drive the variations of SOC and SON have yet to be estimated. We hypothesized that coastal reclamation modifies soil microbial biomass, as well as community composition by altering soil nutrient substrates, e.g., SOC, water-soluble organic carbon (WSOC), SON and soil physiochemical properties, which in turn affects SOC and SON decomposition and accumulation following the conversion of coastal wetland to aquaculture pond, farmland, and town construction land. To test this, we examined the microbial biomass and community composition of the soils through phospholipid fatty acids (PLFA) analysis, and a chloroform fumigation-extraction method. We analyzed SOC, WSOC, SON, soil moisture, salinity, pH, and bulk density (BD) in reclaimed coastal aquaculture pond, wheat and oilseed rape fields, and town construction land by comparing them with an adjacent natural *Phragmites australis* wetland. The objectives of this study were to: (1) evaluate whether the responses of soil microbial biomass and community composition to coastal reclamation varied between different land use types; (2) identify the most important driving factors for causing shifts in soil microbial biomass and community composition following coastal reclamation.

## Results

### Plant and soil physicochemical characteristics

The wheat and oilseed rape fields exhibited higher aboveground biomass compared with the *P. australis* wetland (Table 1). The wheat field showed higher belowground biomass in contrast to the oilseed rape field (Table 1). The total biomass was highest in wheat field followed by oilseed rape field, compared to the *P. australis* wetland (Table 1). The soil/sediment moisture was highest in the aquaculture pond followed by the wheat field and oilseed rape field, in comparison with the *P. australis* wetland and town construction land (Table 1). Soil/sediment pH was highest in the *P. australis* wetland and lowest in the aquaculture pond (Table 1). Soil/sediment salinity in the *P. australis* wetland and aquaculture pond was significantly (*P* < 0.05) higher than that of the wheat field, oilseed rape field, and town construction land (Table 1). The highest soil/sediment bulk density was observed in the oilseed rape field between land use types (Table 1). The concentrations of SOC, WSOC, and SON in the aquaculture pond, wheat field, oilseed rape field, were significantly (*P* < 0.05) higher than those in the *P. australis* wetland (Table 1). Town construction land revealed lower concentrations of SOC, WSOC, and SON compared to the *P. australis* wetland (Table 1).

**Table 1:** Soil physiochemical properties of different land use types following coastal reclamation in the Eastern coastal zone of China.

Characteristics	Land use types					
	*P. australis* wetland	Aquaculture pond	Wheat field	Oilseed rape field	Town construction land	*P-*value
Moisture (%)	25.55 ± 0.37^c^	49.87 ± 1.11^a^	31.80 ± 1.03^b^	30.12 ± 1.20^b^	22.33 ± 0.07^d^	< 0.001
pH	8.83 ± 0.06^a^	7.44 ± 0.05^e^	7.90 ± 0.06^d^	8.17 ± 0.02^c^	8.60 ± 0.01^b^	< 0.001
Salinity (%)	0.53 ± 0.03^a^	0.31 ± 0.04^b^	0.07 ± 0.02^d^	0.18 ± 0.01^c^	0.03 ± 0.01^d^	< 0.001
BD (g cm^–3^)	1.31 ± 0.03^b^	1.07 ± 0.01^c^	1.24 ± 0.05^b^	1.51 ± 0.08^a^	1.21 ± 0.02^bc^	< 0.001
SOC (g kg^–1^)	3.43 ± 0.33^d^	15.07 ± 0.36^a^	9.50 ± 0.24^b^	6.90 ± 0.29^c^	1.86 ± 0.23^e^	< 0.001
WSOC (mg kg^–1^)	82.40 ± 2.55^d^	125.00 ± 1.93^a^	107.25 ± 0.73^b^	98.21 ± 2.74^c^	57.64 ± 2.07^e^	< 0.001
SON (g kg^–1^)	0.24 ± 0.02^d^	1.40 ± 0.05^a^	0.97 ± 0.02^b^	0.68 ± 0.02^c^	0.22 ± 0.03^d^	< 0.001
AB (g m^–2^)	1276 ± 120^b^	–	2253 ± 66^a^	2335 ± 88^a^	–	< 0.01
BB (g m^–2^)	982 ± 224^ab^	–	1351 ± 101^a^	521 ± 101^b^	–	< 0.05
TB (g m^–2^)	2258 ± 184^c^	–	3604 ± 57^a^	2855 ± 167^b^	–	< 0.01

Different superscript lower case letters indicate statistically significant differences at the α = 0.05 level between land use types.

*BD* bulk density, *SOC* soil organic carbon, *WSOC* soil water-soluble organic carbon, *SON* soil organic nitrogen, *AB* aboveground biomass, *BB* belowground biomass, *TB* total biomass.

### Soil microbial biomass and community composition

**Figure 2:**
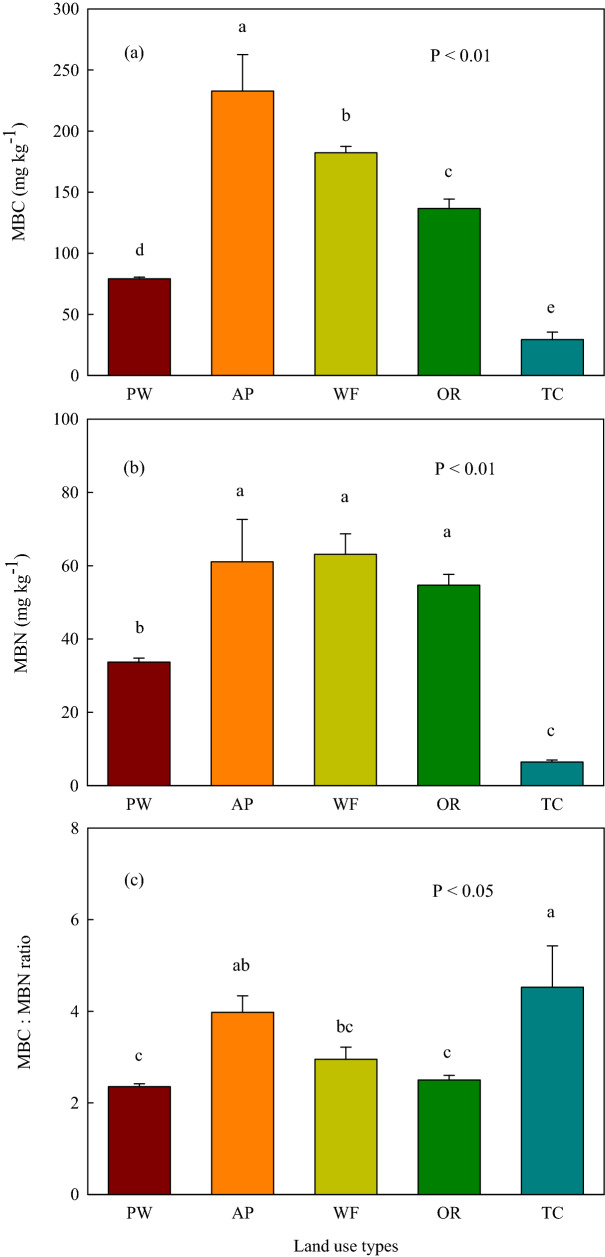
(**a**) Soil microbial biomass carbon (MBC), (**b**) Soil microbial biomass nitrogen (MBN) and (**c**) the MBC:MBN ratio of different land use types following coastal reclamation in the Eastern coastal zone of China. Different letters over the bars indicate statistically significant differences at α = 0.05 level between land use types. *PW* *Phragmites australis* wetland, *AP* aquaculture pond, *WF* wheat field, *OR* oilseed rape field, *TC* town constructive land. Statistically significant differences in this figure were carried out with SPSS statistical software (Version 24.0, URL link: https://www.ibm.com/products/spssstatistics?lnk=STW_US_STESCH_P1_BLK&lnk2=trial_SPSSstat&lot=1&pexp=def&psrc=none&mhsrc=ibmsearch_a&mhq=spss).

The soil/sediment microbial biomass carbon (MBC) concentration was highest in the aquaculture pond, followed by that of the wheat and oilseed rape fields, relative to the *P. australis* wetland and the town construction land (Fig. 2a). The aquaculture pond, wheat and oilseed rape fields revealed higher microbial biomass nitrogen (MBN) concentration in contrast to the *P. australis* wetland and the town construction land (Fig. 2b). The lowest MBC and MBN concentrations were observed in the town construction land between land use types (Figs. 2a,b). The soil/sediment MBC:MBN ratio was highest in the town construction land between land use types (Fig. 2c). The *P. australis* wetland and oilseed rape field showed the lowest soil/sediment MBC:MBN ratio between land use types (Fig. 2c).

**Figure 3:**
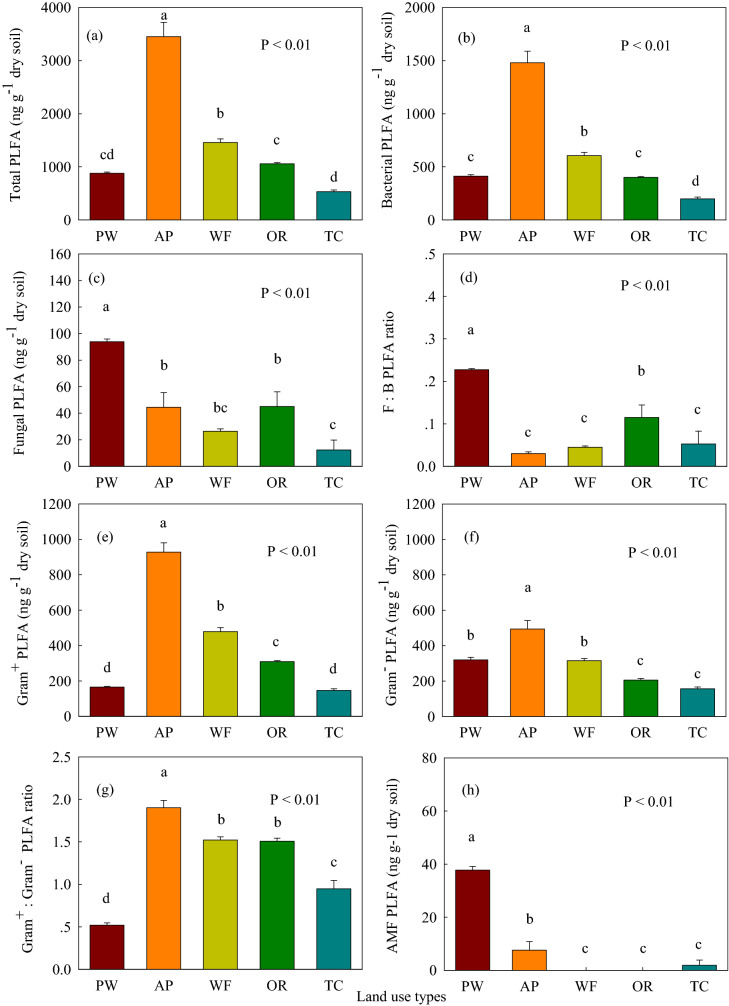
(**a**) Soil total phospholipid fatty acids (PLFA), (**b**) Bacterial PLFA, (**c**) Fungal PLFA concentrations; (**d**) Fungal:Bacterial (F:B) PLFA ratio; (**e**) Gram^–^ PLFA, (**f**) Gram^+^ PLFA concentrations, (**g**) Gram^+^:Gram^–^ PLFA ratio and (**h**) the arbuscular mycorrhizal fungal (AMF) PLFA concentrations of different land use types following coastal reclamation in the Eastern coastal zone of China. Different letters over the bars indicate statistically significant differences at α = 0.05 level between land use types. See Fig. 2 for abbreviations. Statistically significant differences in this figure were carried out with SPSS statistical software (Version 24.0, URL link: https://www.ibm.com/products/spssstatistics?lnk=STW_US_STESCH_P1_BLK&lnk2=trial_SPSSstat&lot=1&pexp=def&psrc=none&mhsrc=ibmsearch_a&mhq=spss).

**Figure 4:**
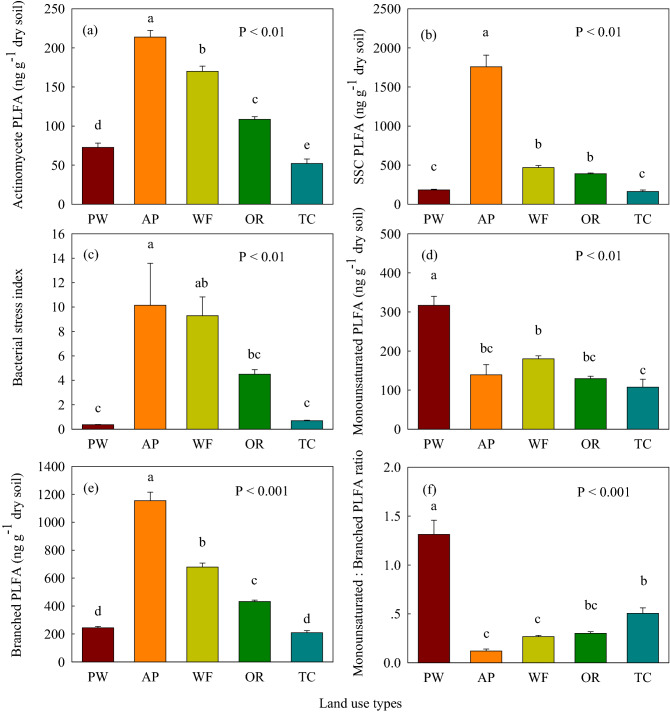
(**a**) Soil actinomycete phospholipid fatty acids (PLFA), (**b**) Saturated straight-chain (SSC) PLFA, (**c**) Bacterial stress index, (**d**) Soil monounsaturated PLFA, (**e**) Branched PLFA concentrations, (**f**) Monounsaturated:branched PLFA ratio of different land use types following coastal reclamation in the Eastern coastal zone of China. Different letters over the bars indicate statistically significant differences at α = 0.05 level between land use types. See Fig. 2 for abbreviations. Statistically significant differences in this figure were carried out with SPSS statistical software (Version 24.0, URL link: https://www.ibm.com/products/spssstatistics?lnk=STW_US_STESCH_P1_BLK&lnk2=trial_SPSSstat&lot=1&pexp=def&psrc=none&mhsrc=ibmsearch_a&mhq=spss).

The total soil/sediment PLFA content in the aquaculture pond increased 1.37–5.49-fold, compared to the *P. australis* wetland, wheat and oilseed rape fields, and town construction land (Fig. 3a). The contents of total, bacterial, gram-positive (gram^+^) bacterial, actinomycete, and branched PLFA were highest in the aquaculture pond followed by the wheat and oilseed rape fields, the *P. australis* wetland, and town construction land (Figs. 3 and 4). The contents of soil fungal, monounsaturated, and arbuscular mycorrhizal fungal (AMF) PLFA were highest in the *P. australis* wetland between land use types (Figs. 3 and 4). The lowest total, bacterial, fungal, gram^+^ bacterial, actinomycete PLFA contents were found in the town construction land (Figs. 3 and 4a). The soil/sediment gram-negative (gram^–^) bacterial PLFA content in the aquaculture pond was significantly (*P* < 0.05) higher than that in *P. australis* wetland, wheat and oilseed rape fields, and town construction land (Fig. 3f). The soil/sediment gram^–^ bacterial PLFA content in the *P. australis* wetland and wheat field was significantly (*P* < 0.05) higher than that in oilseed rape field and town construction land (Fig. 3f). The saturated straight-chain (SSC) PLFA content was highest in the aquaculture pond between land use types (Fig. 4b). The contents of actinomycete and SSC PLFA in wheat and oilseed rape fields was significantly (*P* < 0.05) higher than that in *P. australis* wetland and town construction land (Fig. 4a,b).

The highest soil fungal:bacterial (F:B) PLFA ratio was observed in the *P. australis* wetland between land use types (Fig. 3d). The soil F:B PLFA ratio in aquaculture pond, wheat field and town construction land was significantly (*P* < 0.05) lower than that in oilseed rape field (Fig. 3d). The gram^+^:gram^–^ PLFA ratio was highest and lowest in the aquaculture pond and the *P. australis* wetland, respectively (Fig. 3g). The gram^+^:gram^–^ PLFA ratio in wheat and oilseed rape fields was significantly (*P* < 0.05) higher than that in town construction land (Fig. 3g). The *P. australis* wetland and town construction land exhibited a higher monounsaturated:branched PLFA ratio, relative to the aquaculture pond and the wheat field (Fig. 4f). The bacterial stress index was highest in the aquaculture pond followed by the wheat and oilseed rape fields, and town construction land, which was lowest in the *P. australis* wetland (Fig. 4c).

### Relationships between soil microbial communities and soil properties

**Figure 5:**
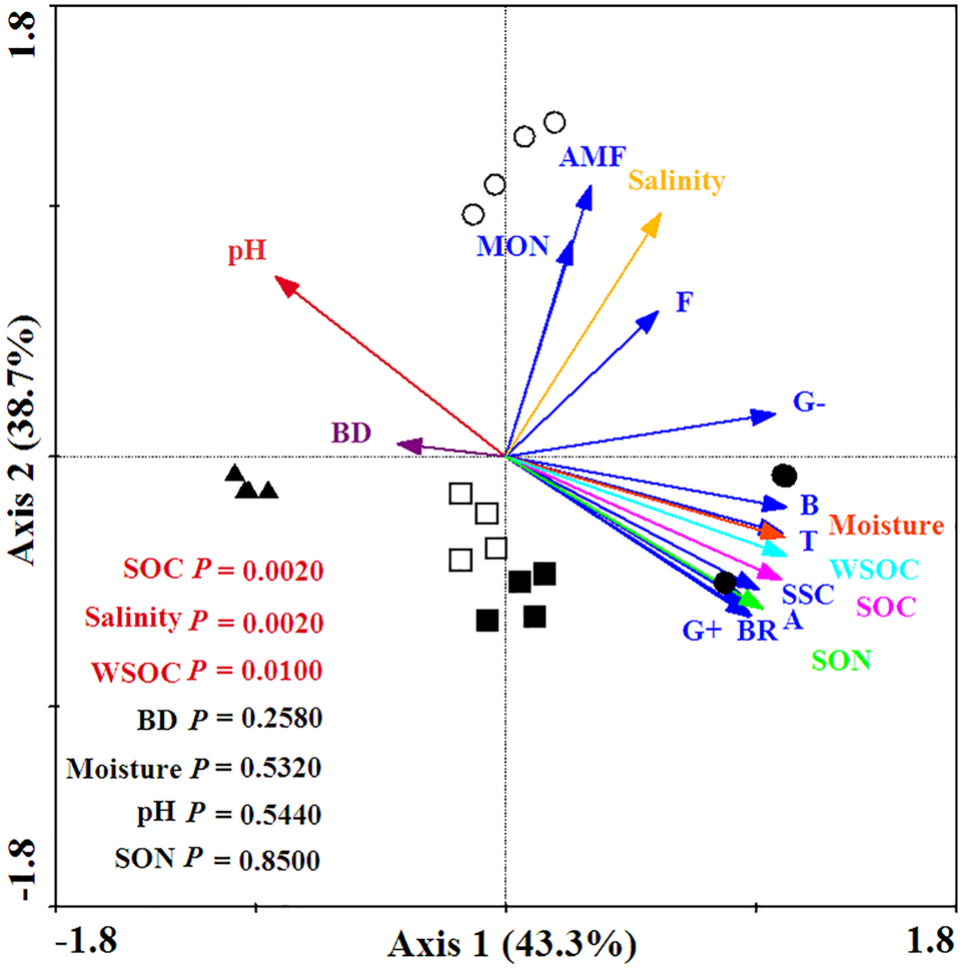
RDA results of PLFA in the soil samples and environmental variables. The explanatory variables are show via different arrows: PLFA profiles are solid blue arrows: total PLFA (T); bacterial PLFA (B); fungal PLFA (F); gram-positive bacterial PLFA (G^+^); gram-negative bacterial PLFA (G^–^); arbuscular mycorrhizal fungal PLFA (AMF); actinomycete PLFA (A); saturated straight-chain PLFA (SSC), monounsaturated PLFA (MON); branched PLFA (BR); and environmental variables are the solid colored arrows: moisture, pH, salinity, bulk density (BD), soil organic carbon (SOC), soil water-soluble organic carbon (WSOC), soil organic nitrogen (SON). Open circles represent *P. australis* soil, filled circles represent the sediment of the aquaculture pond, filled squares represent the soil of the wheat field, open squares represent the soil of oilseed rape field, filled triangles represent the soil of the town constructive land. The statistical significance of the RDA was tested using the Monte Carlo permutation test (499 permutations; *P* < 0.05). Figure was carried out with CANOCO software (Version 4.5, URL link: http://canoco.software.informer.com/4.5/).

Seven soil property variables that were present in the ordination explained 87.2% of the total variability of the PLFA (Fig. 5). The PLFA variations were significantly (*P* < 0.05) related to SOC (*F* = 13.05, *P* = 0.0020), salinity (*F* = 33.20, *P* = 0.0020), WSOC (*F* = 4.29, *P* = 0.0100) (Fig. 5). Pearson's correlation analysis indicated that MBC, MBN, total PLFA, bacterial, gram^+^ bacterial, gram^−^ bacterial, actinomycete, saturated straight-chain, and branched PLFA had obviously positive correlations with soil moisture, SOC, WSOC, and SON, which had a negative correlation with soil pH (Table 2). Soil AMF PLFA was highly related to soil salinity and pH (Table 2). The soil F:B PLFA ratio was inversely associated with the SOC and SON (Table 2). The soil gram^+^:gram^−^ PLFA ratio was highly correlated with soil moisture, SOC, WSOC, and SON (Table 2). However, there was a significant negative correlation between the gram^+^:gram^−^ PLFA ratio and soil pH (Table 2). The soil monounsaturated:branched PLFA ratio had a negative correlation with soil moisture (Table 2).

**Table 2:** Correlation analysis of soil physiochemical properties and microbial communities between land use types following coastal reclamation in the Eastern coastal zone of China.

	Moisture	pH	Salinity	BD	SOC	WSOC	SON
MBC	0.863**	–0.890**	0.109	–0.300	0.937**	0.912**	0.934**
MBN	0.626**	–0.690**	0.146	0.049	0.752**	0.830**	0.754**
MBC:MBN ratio	0.147	–0.171	–0.370	–0.525*	0.060	–0.178	0.087
Total PLFA	0.949**	–0.858**	0.222	–0.542*	0.929**	0.816**	0.895**
Bacterial PLFA	0.965**	–0.831**	0.269	–0.559*	0.917**	0.811**	0.876**
Fungal PLFA	0.034	0.280	0.887**	0.107	–0.081	0.061	–0.181
Fungal:Bacterial (F:B) PLFA ratio	–0.396	0.643**	0.677**	0.355	–0.496*	–0.305	–0.570**
Gram^+^ bacterial PLFA	0.969**	–0.926**	0.071	–0.526*	0.967**	0.858**	0.951**
Gram^−^ bacterial PLFA	0.833**	–0.651**	0.504*	–0.523*	0.799**	0.744**	0.733**
Gram^+^ : Gram^−^ PLFA ratio	0.769**	–0.919**	–0.367	–0.226	0.843**	0.777**	0.885**
AMF PLFA	–0.163	0.508*	0.889**	–0.001	–0.283	–0.171	–0.390
Actinomycete PLFA	0.897**	–0.929**	0.001	–0.413	0.968**	0.919**	0.966**
Saturated straight-chain PLFA	0.965**	–0.848**	0.178	–0.545*	0.902**	0.764**	0.872**
Monounsaturated PLFA	–0.184	0.403	0.774**	0.062	–0.215	–0.046	–0.296
Branched PLFA	0.959**	–0.938**	0.046	–0.505*	0.976**	0.877**	0.964**
Monounsaturated: Branched PLFA ratio	–0.539**	0.777**	0.651**	0.172	–0.635**	–0.489*	–0.710**

*MBC* microbial biomass carbon, *MBN* microbial biomass nitrogen, *PLFA* phospholipid fatty acids, *Gram*^+^ gram-positive, *Gram*^*–*^ gram-negative, *AMF* arbuscular mycorrhizal fungal. See Table 1 for abbreviations.

*P < 0.05; **P < 0.01.

## Discussion

Coastal reclamation enhanced the total microbial biomass (MBC, MBN and total PLFA) (Figs. 2 and 3a), and the quantities of vast majority of microbial community composition following the conversion of *P. australis* wetland to aquaculture pond, wheat, and oilseed rape fields (Figs. 3 and 4). Whereas, the MBC, MBN, bacterial, fungal, gram^–^ bacterial, AMF, actinomycete, and monounsaturated PLFA substantially decreased following the conversion of *P. australis* wetland to town construction land (Figs. 2, 3 and 4). These variation trends of soil microbial communities following coastal reclamation was in according with the results of our previous study showing that coastal reclamation enhanced the accumulation of soil total, labile and recalcitrant organic C and N following conversion of *P. australis* salt marsh into fishpond, wheat and rapeseed fields^21^. Whereas, coastal reclamation decreased the sequestration of soil total, labile and recalcitrant organic C and N following conversion of *P. australis* salt marsh into town construction land^21^. Previous studies reported that the SOC and SON concentrations were determined by organic detritus input, sequestered C and N via bio-chemical and physical processes, loss of organic C and N through SOM decomposition, and erosion and leaching^22^. Chen et al*.*^23^ showed that approximately 30% of the fish food introduced into aquaculture pond was not consumed, which eventually settled into the sediment of aquaculture pond through a series of decomposition processes in the Jiangsu coast. It was reasoned that SOC, WSOC, and SON were highest in the aquaculture pond between land use types, that were largely due to the substantial amount of organic detritus (e.g., organism feces, feed remnants, and partial residual bodies) inputting into the sediment of the aquaculture pond, and ultimately promoted sediment organic C and N sequestration in the aquaculture pond (Table 1)^21,23^. In addition, sediment in the aquaculture pond was immersed in water which provided an anaerobic environment in the sediment. We deduced that high sediment moisture and anaerobic environment in the aquaculture pond were beneficial for sediment organic C and N accumulation over the long-term (Table 1)^21,24^, as SOM accumulated under anaerobic and/or waterlogged conditions (e.g., aquaculture pond) exhibited a lower decomposition rate^25^.

High alkalinity and salinity are basic features of coastal wetlands^20^, which are the primary limiting factors for agricultural production in coastal zones^5,26^. Grybos et al*.*^27^ reported that high soil pH can lead to insufficient nutrients for crop growth owing to promoting the immobilization of manganese, iron, and zinc in soils. Krishnamoorthy et al*.*^28^ documented that high soil salinity severely restricted plant growing, which caused physiological drought to plants, cell toxicity, and nutrient imbalance for crops. Currently, fresh water irrigation has been regarded as a very effective measure to dealkali and desalinate the soil to accommodate the growth of crops following the reclamation of coastal wetlands^5,20,21^. In this study, we found that SOC and SON levels greatly increased following conversion of *P. australis* salt marsh into wheat and rapeseed fields (Table 1). This result is consistent with previous studies, which revealed that reclaimed farmlands effectually promoted SOC and SON sequestration by altering hydrological regimes from ditch drainage, diking, and irrigation, and lower soil pH and salinity relative to coastal wetlands^11,21^. It was inferred that greatly decreased soil pH and salinity, and increased inputs of aboveground biomass, as well as the application of chemical fertilizers contributed to greater SOC and SON accumulation in the wheat and oilseed rape fields compared to *P. australis* wetland (Table 1)^21^. Conversely, SOC, WSOC, and SON levels were lowest in town construction land (Table 1), which may have been owing to the loss of vegetative cover and without exogenous organic detritus entering the soil.

Coastal reclamation greatly shifted soil/sediment microbial biomass and community composition (Figs. 2, 3 and 4). In this study, the redundancy analysis (RDA) clearly showed that the variations in soil microbial community were the most intimately related to SOC, salinity, and WSOC (Fig. 5), which further demonstrated that soil nutrient substrates (e.g., SOC, WSOC, and SON) were the overarching driving factors for soil microbial communities^29^, especially for soil bacteria and fungi^30, 31^, as they provided a great quantity of available nutrients for soil microbes^32^, and played crucial roles in altering the composition of microbial communities for resource competition^33^. Additionally, previous studies demonstrated that high soil salinity has a considerable effect on growth^34^, quantity and structure^17^ of soil microbes, as well as inhibited extracellular enzyme activity through altering the habitat of soil microbes^35^. Aside from SOC, soil salinity, and WSOC, the Pearson's correlation analysis indicated that the total and the vast majority of soil microbial compositions were highly correlated with soil moisture, which were significantly negatively related to soil pH (Table 2). This finding was supported by previous studies suggesting that soil pH and moisture played vital roles in altering soil microbial biomass and community composition^15,18,19^. Thus, we extrapolated that greatly increased soil/sediment microbial biomass (MBC, MBN and total PLFA), as well as various microbial community composition (i.e., gram-positive bacterial, actinomycete, saturated straight-chain, and branched PLFA) following the conversion of *P. australis* wetland to aquaculture pond, wheat, and oilseed rape fields were primarily attributed to the higher level of soil nutrient substrates, and decreased soil salinity and pH which lifted the restriction of high salinity and alkalinity on the growth of soil microbial communities in aquaculture pond, wheat, and oilseed rape fields (Tables 1 and 2; Figs. 2, 3, and 4).

Among soil microbes, AMF community plays a crucial role in enhancement of nutrient uptake and the tolerance of their host plants to various environmental stresses^36,37^. Interestingly, we found that the quantity of AMF PLFA substantially reduced following the conversion of *P. australis* wetland to other soil land use types (Fig. 3h). This result was supported by Cui et al*.*^37^ exhibiting that coastal reclamation negatively affects AMF community structure and diversity in coastal saline-alkaline lands during the past 30 years of reclamations. Previous studies demonstrated that soil salinity and pH were the dominant factors driving structure and the distribution of soil AMF community^37,38^. Our Pearson's correlation analysis displayed that soil AMF PLFA was highly related to soil salinity and pH (Table 2). Coastal wetland is the buffer zone between the sea and land, and it is characterized by its high salinity, high pH, low nutrient substrates, varied temperatures, and an unstable sandy substrate^39^. It was deduced that AMF community played a vital role in the coastal wetlands ecosystem, and the most enriched AMF community in *P. australis* wetland can provide more nutrient elements (e.g., N and P) for *P. australis* community, and assist *P. australis* community to adapt oligotrophic and extreme environment with multiple stresses (Table 1 and Fig. 3h)^36,37^. When soil properties tended to be stable and the needed nutrients for plant growth raised (Table 1), the role of AMF community altered and their quantity became less (Fig. 3h)^37^.

The F:B PLFA ratio is considered to be a key index for evaluating the responses of fungal and bacterial biomass to environmental variabilities^32,40^. Interestingly, coastal reclamation significantly (*P* < 0.05) increased soil bacterial PLFA following the conversion of *P. australis* wetland to aquaculture pond and wheat field (Fig. 3b), whereas soil fungal PLFA and the F:B PLFA ratio substantially decreased following coastal reclamation (Fig. 3c,d). Previous studies have documented that the availability of soil nutrient is the dominating factor that impacts the F:B PLFA ratio^32^. The responses of soil bacterial and fungal communities to the availability of soil nutrient can entirely differ^32,41–43^. Generally, soil bacterial communities with higher organic matter inputs, combined with plentiful available nutrients are more remarkably abundant compared with fungal communities^32,43^. Soil fungal communities have the capacity to degrade more recalcitrant organic materials and prefer nutrient-poor environments^41,42^. Wang et al*.*^44^ documented that the F:B PLFA ratio has significant negative correlation with soil nutrient availability. Thus, the highest quantity of soil fungal PLFA and F:B PLFA ratio in the *P. australis* wetland may have been primarily attributed to low nutrient availability which promoted the growth of fungi rather than bacteria, as bacteria favor nutrient-rich conditions^45^, while fungi prefer conditions with low nutrient levels (Fig. 3c,d)^46^.

The gram^+^:gram^–^ PLFA ratio is recognized as an important indicator for microbial community structures and ecological functions^47^. In this study, the gram^+^:gram^–^ PLFA ratio ranged from 0.52 to 1.90 between land use types (Fig. 3g), which exhibited that gram^−^ bacteria dominated in the *P. australis* wetland, and gram^+^ bacteria dominated in the reclaimed land use types. Coastal reclamation greatly raised gram^+^:gram^–^ PLFA ratio following the conversion of *P. australis* wetland to other land use types (Fig. 3g). Previous studies have reported that gram^+^ bacteria are considered as oligotrophic K-strategists^44,48^, which prefer to utilize recalcitrant soil C (e.g., SOM-derived C) as an energy source, with slow growing rates^49^. Conversely, gram^−^ bacteria favor soils with easily degradable organic substances (e.g., plant materials and fungal exudates) as carbon sources^42^, which are viewed as copiotrophic r-strategists^44,48^. However, our the Pearson's correlation analysis showed that the gram^+^:gram^−^ PLFA ratio was positively correlated with SOC, WSOC, and SON (Table 2), which was consistent with the results presented by Xu et al*.*^47^ and Luo et al.^50^. Further, earlier study reported that soil pH plays a crucial role in modifying the composition of bacterial communities^51^. Rousk et al*.*^15^ confirmed that gram^–^ bacteria biomass increases, while gram^+^ bacteria biomass decreases, in response to higher soil pH. It may be presumed that the lowest gram^+^:gram^–^ PLFA ratio in the *P. australis* wetland may be partly the result of the highest soil pH in the *P. australis* wetland, which promoted gram^–^ bacteria growth (Fig. 3g)^15,52^. This deduction was supported by our finding that the gram^+^:gram^−^ PLFA ratio exhibited a significant negative correlation to soil pH (Table 2). Typically, the bacterial stress index may be employed to indicate the physiological status of gram^–^ bacteria communities^53^. A high bacterial stress index represents a slow growth phase a slow rate of growth and slow turnover of gram^–^ bacteria, as the result of being affected by various stresses, such as low pH^54,55^. The bacterial stress index was highest in the aquaculture pond (Fig. 4c), which suggested that a low rate of growth and slow turnover of gram^–^ bacteria was observed in the aquaculture pond relative to gram^+^ bacteria due to high stress from the lowest soil pH (Table 1; Figs. 3g and 4c).

The soil monounsaturated:branched PLFA ratio can indicate the relative ratio of aerobic to anaerobic microbes^32,54,56^. The soil monounsaturated:branched PLFA ratio was highest in the *P. australis* wetland (Fig. 4f), which implied that *P. australis* wetland possessed the highest proportion of aerobic microbes between land use types. This was tightly associated with the lowest soil moisture and high soil aeration in the *P. australis* wetland (Table 1). Contrarily, the lowest soil monounsaturated:branched PLFA ratio was observed in the aquaculture pond (Fig. 4f), which indicated that it had the highest proportion of anaerobic microbes between land use types. This result was likely caused by high anaerobic state (i.e., flooded conditions) of the sediment in the aquaculture pond (Table 1), which is beneficial for the growth of anaerobic microbes (Fig. 4e). The highest proportion of anaerobic microbes were accompanied by high anaerobic environment can slow down the decomposition of SOM (Fig. 4e)^24^, and promote SOC and SON sequestration in the aquaculture pond (Table 1)^32^.

In conclusion, this study emphasized the shifts in soil microbial biomass and community composition in *P. australis* wetland that have been converted to different land use types in the Eastern coastal zone of China. Our study suggested that coastal reclamation altered the soil microbial biomass and community composition through the modification of soil nutrient substrates (SOC, WSOC, and SON) and physiochemical properties (e.g., soil salinity, pH and moisture) of the soil. Coastal reclamation greatly altered the F:B PLFA, gram^+^:gram^–^ PLFA, and monounsaturated:branched PLFA ratios. These changes in microbial community structures were involved in regulation of SOC and SON decomposition and accumulation. This study offers new insights toward a better understanding of the consequences of coastal reclamation to ecosystem processes and functions, as well as the further elucidation of variations in, and drivers of, soil microbial communities.

## Methods

### Study site and sampling

This study was conducted in the Yancheng Yellow Sea coast of Jiangsu Province, China (Fig. 1). Specific sampling transects were located next to the Dafeng Nature Reserve (32°00′–33°15′ N, 120°40′–121°00′ E; Fig. 1). This area has an annual average temperature of 14.4 °C and an annual average precipitation of 1088 mm. The natural vegetations of the Yancheng Yellow Sea coast are listed from sea to inland: *Spartina alterniflora*, *Suaeda salsa*, *Imperata cylindrica*, and *P. australis* communities (Fig. 1)^32^. Over the last century, the wetlands of the Jiangsu coast have undergone intensive reclamation^11^. At present, most of coastal wetlands have been reclaimed and converted to aquaculture ponds, farmlands, and town construction lands, particularly in Dafeng and Sheyang counties (Fig. 1). Wheat (*Triticum aestivum* L.) and oilseed rape (*Brassica campestris* L.) fields are widely distributed along the middle Jiangsu coast. *P. australis* wetlands are the easiest to reclaim to other land use types due to their growing further inland, as they are farthest from the sea, relative to *S. alterniflora*, *S. salsa*, and *I. cylindrica* salt marshes (Fig. 1)^21^.

In June 2016, four sample transects of 40 m × 40 m were selected in each land use type, i.e., *P. australis* wetland (control), aquaculture pond, wheat field, oilseed rape field, and town construction land (Fig. 1), respectively, where there was a distance of 100 m between any two adjacent sample transects in each land use type. Satellite images (1975, 1991, 2000, 2006, 2010, and 2013 year) from the Landsat Thematic Mapper and historical records of the Yancheng Yellow Sea coast of Jiangsu Province were analyzed to identify the reclamation time of land use types and the types of natural salt marsh prior to coastal reclamation in the sampling region. The aquaculture ponds, wheat fields, and oilseed rape fields in the sample transects had been reclaimed for approximately 25 years, and were originally *P. australis* wetlands^21^. The aquaculture ponds in the sample transects were mainly used for raising silver carps. The wheat fields in the sample transects were used to plant winter wheats, and the oilseed rape fields in the sample transects were planted with winter oilseed rapes, and their yields or biomass had reached the maximum due to the sampling time is the ripe season for winter wheats and winter oilseed rapes. The town construction lands in the sample transects had been established for 6 years, which suffered continual coastal reclamation from *P. australis* wetlands in 1975, to aquaculture ponds in 1991, and were further converted to town construction lands in 2010^21^. The town construction lands in the sample transects were selected in the open spaces around the buildings of the urban construction, and the open spaces were the lands rather than cement or brick floors, and the open spaces were little vegetation cover due to intensive artificial disturbance. Due to the significant extent of *P. australis* wetlands being reclaimed to farmlands, aquaculture ponds, and town construction lands, only a small area of *P. australis* wetland remained in the sampling region (Fig. 1). For this study, we randomly selected three 2 m × 2 m plots in each transect, and three sites were selected for the collection of soil samples from each plot. Subsequently, soil samples from each plot were thoroughly mixed to yield a final soil sample. We randomly established three 0.5 m × 0.5 m quadrats to gather all aboveground plant materials and dug three soil blocks (0.15 m long × 0.15 m wide × 0.30 m deep) to gather all of the roots from each transect of the *P. australis* wetland, wheat field, and oilseed rape field.

### Analysis of plant and edaphic properties

Each root-sampling block was put through a 0.15 mm sieve and repeatedly flushed with water; the roots remaining in the sieve were then collected^9^. The aboveground plant materials and roots were carefully cleaned and oven-dried at 65 °C to a constant weight to determine the plant biomass. The soil BD was determined using a cutting ring method. Fresh soil subsamples were oven-dried at 105 °C to a constant weight to measure the soil moisture^32^. Plant debris, soil fauna, and rocks in the soil samples were removed, which were then fully mixed and separated into three subsamples. The first subsample was air-dried and sifted using 1 mm sieve to analyze the soil pH, salinity, SOC, and SON. The second subsample was sifted using a 2 mm sieve and stored at 4 °C to examine WSOC, MBC and MBN. The third subsample was sifted using a 2 mm sieve and stored at –80 °C after freeze-drying and was used to determine the PLFA. The soil pH was determined in a 1:2.5 soil to water suspension using a digital pH meter. The soil salinity was determined in a 1:5 soil to water suspension. The SOC and SON concentrations were quantified using a CN elemental analyzer (Vario Micro CHNS analyzer, Germany), where prior to determination, the soil samples were added to 1 M HCl to eliminate inorganic C and N. The determination of WSOC proceeded according to the technique described by Yang et al.^32^.

### Analyses of soil microbial biomass and community composition

The soil MBC and MBN were measured via chloroform fumigation-extraction^57^. Fresh soil samples (25 g dry weight equivalent for soil microbial biomass) were fumigated for 48 h with ethanol-free chloroform at 25 °C in the dark. Additional aliquots of fresh soil were employed as unfumigated controls. Both the fumigated and unfumigated samples were then extracted with 100 mL of 0.5 M K_2_SO_4_ by agitating for 30 min at 200 rpm using a reciprocal shaker, after which the K_2_SO_4_ extracts were passed through 0.45 μm filters. Soil extractable organic C and total N by K_2_SO_4_ extracts was quantified with a Liqui TOCII analyzer and the Kjeldahl method, respectively. MBC and MBN were calculated according to the equation: MBC = Ec/0.38, MBN = En/0.54, where Ec and En are organic C and total nitrogen (TN) extracted from fumigated soil, subtracted organic C, and TN extracted from unfumigated soil, respectively.

The PLFA analysis was used to determine soil microbial biomass and community composition^58^. The PLFA was determined in accordance with the procedure previously described by Bossio and Scow^58^ and Yang et al*.*^32^. Briefly, 8 g of a dry weight-equivalent of the soil subsamples was extracted in 23 mL of a chloroform: methanol: phosphate buffer mixture (1:2:0.8, v/v/v). The extraction was decanted into a separatory funnel and added to 12 mL of CHCl_3_ and 12 mL of phosphate buffer following centrifugation. The separatory funnel was shaken for 2 min., and the extracts were layered overnight. The CHCl_3_ layer was collected and dried under N_2_ at 32 °C, whereas the lipids were re-dissolved in chloroform and fractionated on a 0.5-g silica gel solid-phase extraction column (Supelco, Bellefonte, PA). Neutral and glycol lipids were eluted by 5 mL of CHCl_3_ and 10 mL of acetone. Polar lipids were collected via 5 mL of methanol, dried under N_2_ at 32 °C, and then subjected to a mild-alkali methanolysis to recover the PLFA as methyl esters. The samples were re-dissolved in 200 mL of hexane solvent containing nonadecanoic acid methyl ester (19:0) as an internal standard. The samples were analyzed using a Hewlett-Packard 6890 Gas Chromatograph equipped with an Ultra 2-methylpolysiloxane column with N_2_ as the carrier gas, and H_2_ and air to support the flame. A 2-μL injection of the above dilution with a 1:50 split was employed at 250 °C for the injector and 300 °C for the detector. The oven temperature was ramped from 170 °C to 300 °C at 5 °C/min^–1^ and was maintained for 12 min.. The peaks were identified using bacterial fatty acid standards and MIDI peak identification software (Version 6.2, MIDI Inc., Newark, DE, US, URL link: http://midi-inc.com/index.html). The quantities (ng g^–1^ dry soil) of the PLFA in each sample were analyzed using an internal standard (19:0, 5 μg mL^–1^). The quantities of the PLFA in each sample were expressed as ng PLFA g^–1^ dry soil and were used to estimate the microbial biomass. The bacterial biomass was indicated by the biomarkers i14:0, i15:0, a15:0, 15:0, i16:0, i17:0, a17:0, 17:0, cy17:0, 14:1ω5c, 15:1ω6c, 16:1ω7c, and 18:1ω7c^44,56,58,59^. Indicators of gram^+^ bacteria included i13:0, i14:0, i15:0, a15:0, i16:0, a16:0, i17:0, and gram^–^ bacteria included 14:1ω5c, 15:1ω6c, 16:1ω7, 16:1ω9c, 17:1ω8c, 18:1ω7c, 12:0 2OH, 15:0 3OH, 16:1 2OH, cy17:0, cy19:0 ω8c, and 18:1ω7c 11-methyl^32,56,60^. The fungal biomass was quantified by the sum of the PLFA 18:1ω9c, 18:2ω6,9c, and 20:1ω9c^59,60^. The AMF biomass was assessed by the PLFA 16:1ω5c^9,56,60^. The 10me 16:0 and 10me 17:0 biomarkers were used as representatives of the Actinomycete biomass^54^. The monounsaturated PLFA was quantified by the sum of 14:1ω5c, 15:1ω6c, 16:1ω5c, 16:1ω7c, 16:1ω9c, 17:1ω8c, 18:1ω7c, 18:1ω9c, and 20:1ω9c^9,54,56^. The sum of i13:0, i14:0, i15:0, a15:0, i16:0, a16:0, i17:0, a17:0, 10me 16:0, 10me 17:0, 12:0 2OH, 15:0 3OH, and16:1 2OH were used as indicators of branched PLFA^54,56,58^. The SSC PLFA was indicated by the biomarkers 12:0, 13:0, 14:0, 15:0, 16:0, 17:0, 18:0, and 20:0^56,58^. The total PLFA of soil microbial communities was calculated by the sum of the fungal PLFA, gram^+^ bacterial PLFA, gram^–^ bacterial PLFA, AMF PLFA, actinomycete PLFA, SSC PLFA, and 20:4ω6,9,12,15c. The F:B PLFA, gram^+^:gram^–^ PLFA, and monounsaturated:branched PLFA ratios were calculated from the above PLFAs. Bacterial stress indexes, indicating the microbial physiological status under environmental stresses, were typically represented by cy17:0:16:1ω7c^9^.

### Statistical analyses

One-way analysis of variance (ANOVA) was employed to analyze the impacts of coastal reclamation on soil and plant characteristics, SOC, SON, microbial biomass, and various types of PLFA using SPSS statistical software (Version 24.0, URL link: https://www.ibm.com/products/spssstatistics?lnk=STW_US_STESCH_P1_BLK&lnk2=trial_SPSSstat&lot=1&pexp=def&psrc=none&mhsrc=ibmse arch_a&mhq = spss). Pearson's correlation analysis was used to evaluate the relationship between the C and N fractions of the soil, and microbial biomass with soil physiochemical properties. Linear regression analysis was performed to determine the relationship between soil C and N, and the soil microbial biomass with plant biomass between the *P. australis* wetland, wheat field, and oilseed rape fields. The relationships between the soil microbial communities (all types of PLFA) and soil properties were conducted using RDA with CANOCO software (Version 4.5, URL link: http://canoco.software.informer.com/4.5/). The statistical significance of the RDA was tested using the Monte Carlo permutation test (499 permutations; *P* < 0.05). The map in the Fig. 1 was generated using the ArcGIS software (Version 9.3, URL link: http://desktop.arcgis.com/zh-cn/desktop/).
